# Supplementary material for: The discovery of novel noncoding RNAs in 50 bacterial genomes
Source: Nucleic Acids Res. 2024 Apr 22;52(9):5152–65. doi: 10.1093/nar/gkae248 (PMC11109978; doi:10.1093/nar/gkae248)
Supplement: gkae248_Supplemental_Files [file gkae248_supplemental_files.zip › Narunsky 50 Genomes RNA Motifs Files.zip!/Publication/54-P_aestuarii/Figures/LRC-54-8-NC_011059_1_IGR_26_643187_643559.sto.pdf]

LRC-54-8-NC_011059_1_IGR_26_643187_643559.sto.r2r

5′-UGAAGGYGCUUG●●UAGG●ACAAGU●CYUU●●Y●●YC●G●●AGGUUAGUGUUGCUGCUU●A●●UGUUY●○○○○○○○○○○○GYR●●●●●●●RRUG●U●G●U●AG●●●○○○○○○○○○○○○○AYR●G○○○○●○YCRUGUUUU●○○GY●UUU●CYGRUYUGU●GUYRARC●CGG●RGGAGG●○●●RARR●●●CYUCYGC●G●U●●UU○○○○○○○○○○○●YCC●C●YUUY●Y●U●○YC●UY●●YY●U●YUU●C●RU●A●●●U○○○○○○○○○○○○○○○○○○○○○○○○○○○○○○○○○○○○○○○○○○○○○○○○○○○○○○○○○○
